# Extended Survival with Pancreatic Carcinosarcoma: A Case Report and Literature Review

**DOI:** 10.3390/curroncol32080470

**Published:** 2025-08-18

**Authors:** Tian Xiao, Claire Browne, Morgan Black, Celia Marginean, Elena Tsvetkova

**Affiliations:** 1 Department of Gastrointestinal Oncology, Princess Margaret Hospital, University Health Network, Toronto, ON M5G 2M9, Canada; 2 Department of Oncology, London Health Sciences Centre, London, ON N6A 5W9, Canadamorgan.black@lhsc.on.ca (M.B.); 3 Department of Pathology, Baylor College of Medicine, Houston, TX 77030, USA

**Keywords:** pancreatic carcinosarcoma, extended survival, multi-disciplinary, chemotherapy, rare tumors

## Abstract

Pancreatic carcinosarcoma is a rare and aggressive malignancy that can mimic pancreatic adenocarcinomas in presentation but often has different disease biology and different responses to conventional treatment for pancreatic adenocarcinoma. Case reports have documented a 5-year overall survival of approximately 13% only if the disease is caught at an earlier stage and is amenable to multi-modality treatment, including surgery, chemotherapy, and radiation. In the advanced stage, treatments do not often provide benefit, and patients may decline rapidly. There are currently no studies demonstrating survival benefits with chemotherapy in patients with metastatic carcinosarcoma, owing to both the rarity and the often late diagnosis of this aggressive entity. We present a case of a 71-year-old male patient diagnosed with metastatic pancreatic carcinosarcoma who received four lines of palliative-intent treatment: gemcitabine and nab-paclitaxel, modified FOLFIRINOX, GTX, and doxorubicin. With careful selection of chemotherapeutic regimen as well as his ability to tolerate four lines of treatment, this resulted in an unprecedented 26-month survival. We also reviewed the literature on the histopathology, diagnosis, and treatment of this rare entity.

## Simple Summary

Pancreatic carcinosarcoma is a very rare and aggressive type of cancer that contains both gland-like (carcinoma) and connective tissue (sarcoma) elements. Because it is so uncommon, doctors do not yet have a standard treatment approach, especially when the disease is already widespread. This case report describes a 71-year-old man with advanced pancreatic carcinosarcoma who was able to undergo four different types of chemotherapy for over two years, an unusually long survival for this diagnosis. His care team carefully chose treatments that targeted both components of his cancer and adapted his care as his condition changed. This case shows that with close monitoring as well as thoughtful and collaborative treatment planning, even patients with rare and advanced cancers may benefit from prolonged survival and quality of life. These findings could help guide future research and clinical decisions for similarly rare and complex cancers.

## 1. Introduction

Pancreatic carcinosarcoma is an extremely rare and aggressive malignant tumor, comprising distinct elements of both sarcomas and carcinomas. These are significantly rarer than pancreatic adenocarcinomas, with documentation in the literature limited to case reports and case series; the largest of these, the Surveillance, Epidemiology, and End Results (SEER) study, analyzed 39 patients [1]. Pancreatic carcinosarcomas are classified by the World Health Organization (WHO) as belonging to the group of “undifferentiated carcinoma of the pancreas” alongside sarcomatoid carcinoma and anaplastic giant-cell carcinoma of the pancreas [2]. The terms “carcinosarcoma” and “sarcomatoid carcinoma” have historically been used interchangeably, further complicating discussion of these distinct entities.

Regarding prognosis, the existing small studies reported to date have only documented treatment of localized disease, with potential survival benefits in those who can receive upfront resection and adjuvant chemotherapy. Excluding a few longer-surviving outliers, these cases reported a median recurrence-free survival of up to 15 months; survival after recurrence was not long [3,4,5], in the range of a few months at best. After recurrence, patients are typically not fit enough to receive disease-targeted therapy, instead relying upon best supportive care.

This study reports a case of de novo metastatic pancreatic carcinosarcoma to the liver, where the patient was fit enough to receive chemotherapy in the metastatic setting. We also performed a literature review of reported cases to establish a therapeutic strategy for metastatic pancreatic carcinosarcoma.

## 2. Materials and Methods

During creation of figures in this manuscript, BioRender was used (https://BioRender.com/teihylh). Additionally, OpenAI’s ChatGPT 4o model was used to help generate the graphical abstract.

## 3. Case Report

A 71-year-old gentleman presented with a two-week history of epigastric discomfort and a 10-pound weight loss. His past medical history was otherwise significant for hypertension, gastroesophageal reflux disease, anxiety, depression, and ulcerative colitis, which had been quiescent for 40 years. His medications included sertraline, clonazepam, lansoprazole, atorvastatin, amlodipine, low-dose aspirin, and Vitamin B complex. His family history was remarkable for a brother with renal cell carcinoma, a daughter with ovarian carcinoma, and a grandson with hepatoblastoma. A hereditary cancer screening panel (ATM (NM_000051.3), BRCA1 (NM_007294.3), BRCA2 (NM_000059.3), CDKN2A (NM_058195.3), CDKN2A (NM_000077.4), EPCAM (NM_002354.2), MLH1 (NM_000249.3), MSH2 (NM_000251.2), MSH6 (NM_000179.2), PALB2 (NM_024675.3), PMS2 (NM_000535.5), STK11 (NM_000455.4), TP53 (NM_000546.5)) was subsequently performed which did not identify any genetic aberrations. His physical examination was unremarkable.

Initial CT evaluation revealed a 4.6 × 3.2 cm unresectable pancreatic uncinate mass with innumerable hypoattenuating lesions throughout both lobes of the liver, highly concerning for liver metastases. A 99M-technetium bone scan revealed a T8 sclerotic lesion suspicious for metastasis. The primary pancreatic cancer encased both the superior mesenteric vein and superior mesenteric artery (SMA), with further compression of the SMA. There was no evidence of intrathoracic metastatic disease.

**Figure 1:**
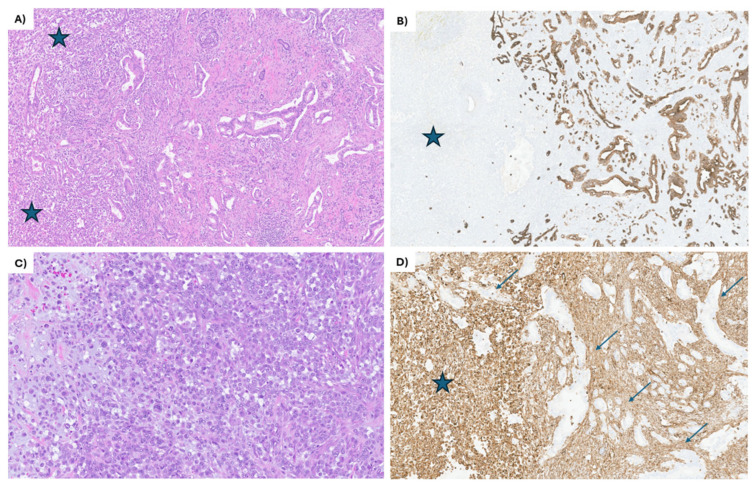
IHC staining of the EUS-guided biopsy of the primary pancreatic head mass. (**A**) The tumor shows 2 distinct components, partially intermingling: the mesenchymal component (blue stars on the left side of the image) and the epithelial component, composed of moderately differentiated ductal adenocarcinoma (on the right side). The ductal adenocarcinoma is composed of angulated glands lined by low cuboidal epithelium. Hematoxylin and eosin, 20×. (**B**) The epithelial component (on the right side) shows strong keratin cytoplasmic staining of the tumor cells, while completely negative in the mesenchymal component (blue star). CK7 immunohistochemical stain, 20×. (**C**) The mesenchymal component is composed almost entirely of large, epithelioid cells, some with pleomorphic nuclei, prominent nucleoli, and numerous atypical mitotic figures. Focally, myxoid stroma is noted (left side of the image). Hematoxylin and eosin, 60×. (**D**) All the tumor cells in the mesenchymal component are strongly positive for vimentin (blue star), while the epithelial component is negative (arrows). Vimentin immunohistochemical stain, 60×.

An endoscopic ultrasound (EUS)-guided biopsy of the pancreatic head mass was performed, which showed a biphasic tumor most consistent with carcinosarcoma. Immunohistochemistry (IHC) was performed and showed the tumor cells were positive for pancytokeratin, BerEp4 (epithelial marker), vimentin (mesenchymal marker), and focally for smooth muscle actin. Additionally, tumor cells were patchy positive for CD68 as well as negative for MelanA, S100, desmin, myogenin, and CD45 (Figure 1). This was reviewed by several local pathologists with expertise in gastrointestinal (GI) and soft tissue pathology. The tumor sample was analyzed with IHC for MLH1, MSH2, MSH6, and PMS2, which demonstrated intact nuclear protein expression for all biomarkers, consistent with mismatch repair (MMR) protein proficient disease. In-house molecular testing by next-generation sequencing (NGS) revealed KRAS (Gly12Val), TP53 (Tyr220Cys), NF2 (Leu127*) classified as Tier I/II, and TSC2 (Arg1795His) (52.9%) as a variant of unknown significance. Unfortunately, none of the identified alterations were targetable. The complete blood count, standard chemistry, liver, and renal function were within normal limits. His CA19-9 was beyond the upper limit of detection (>9999 U/mL at our local institution).

Given the extensive metastatic disease, commencement of palliative chemotherapy was recommended to the patient. Gemcitabine and nab-paclitaxel (Abraxane) were the first-line regimen employed, given the known efficacy of gemcitabine and taxanes in both pancreatic adenocarcinoma and sarcomas. A total of 18 cycles were completed. Treatment was initiated at full dose, and from cycle 8 onwards, a 20% dose reduction was implemented due to anorexia, fatigue, myelosuppression, nausea, and diarrhea.

Treatment started in March 2022, and CA19-9 steadily decreased to a nadir of 239 U/mL at 9 months of uninterrupted chemotherapy. CT of the thorax/abdomen/pelvis showed a marked decrease in size of the pancreatic primary, which, at best response, remained present around the SMA but was no longer well-delineated. All liver metastases demonstrated partial response, and the T8 bone lesion showed evidence of further sclerosis, indicating treatment response.

After 12 months of treatment, there was unfortunately biochemical progression with CA19-9 rising to 306. However, a CT thorax/abdomen/pelvis scan showed ongoing stability, and thus, his treatment continued for another 6 months. At 18 months of total treatment, the CA19-9 had risen to 1396 U/mL. Restaging CT thorax/abdomen/pelvis again showed overall stable disease; a liver lesion grew from 1.3 cm to 1.5 cm but did not meet RECIST 1.1 criteria for progression, and a nonspecific linear band of density in segment 5 was identified. The patient was otherwise asymptomatic. Given the rising CA19-9, which, due to its initial decline in response to treatment, was considered a relevant marker of his disease, it was felt that his disease was slowly progressing. He was offered the options of continuing current therapy with a short interval scan or changing to second-line therapy. Unfortunately, there were no clinical trials accessible to him at that time.

He ultimately chose to switch to second-line modified FOLFIRINOX (leucovorin, 5-fluorouracil, irinotecan, and oxaliplatin), which was started at a 20% dose reduction across all chemotherapeutic agents. His CA19-9 at initiation of second-line therapy was 3427 U/mL. This treatment course was complicated by severe cellulitis, requiring hospital admission, with a 2-week delay in the chemotherapy schedule. He ultimately completed eight cycles over 4.5 months. Second-line therapy was discontinued 21 months after diagnosis due to both biochemical and imaging-identified progression. His CA19-9 rose to 8764 U/mL, and a CT thorax/abdomen/pelvis scan confirmed progression in both the pancreatic head primary (increasing from 0.8 cm to 3.2 cm) and the liver (with an index lesion increasing from 2.0 cm to 2.7 cm and a new 0.7 cm nodule).

Clinically, he remained well with no focal symptoms and a relatively stable bloodwork panel; therefore, he was recommended third-line therapy with GTX (gemcitabine, 750 mg/m^2^ on days 4 and 11; capecitabine, 1000 mg twice daily for 2 weeks; and docetaxel, 30 mg/m^2^ on days 4 and 11) every 3 weeks, with a 20% dose reduction on the docetaxel due to previous neuropathy. At initiation of third-line therapy, his CA19-9 was 8764 U/mL. The treatment initially provided a reasonable response, with his CA19-9 decreasing to 6505 U/mL. Unfortunately, after the third cycle, his CA19-9 rose to >9999 U/mL, with progression on imaging. A CT thorax/abdomen/pelvis scan displayed enlargement of the pancreatic primary to 3.8 × 2.8 cm with extension into the second and third parts of the duodenum, as well as an increase in size of the index liver mass from 2.7 cm to 3.1 × 3 × 3 cm. The patient had also started to develop symptoms of abdominal discomfort, bloating, nausea, and mild fatigue, which were well managed with pain medication and anti-nauseants.

He was clear in his wishes for further treatment and felt his symptoms were well-managed. There was no clear guidance available on further chemotherapy for the carcinoma component of his disease, and it was felt that targeting the sarcoma component was feasible. He qualified for enrolment into a clinical trial (NCT01446744) employing stereotactic ablative radiotherapy (SABR) to all visible sites of disease, which would allow for further systemic therapy at the oncologist’s discretion, but initial workup would take additional time. Considering his worsening symptoms and evident disease progression, and due to the time needed for a trial enrolment process, an urgent start of doxorubicin was favored over enrolment into the SABR clinical trial. A liver biopsy and private-pay extended NGS were also planned but were later canceled by the patient due to progression of symptoms.

A single cycle of weekly doxorubicin at 25 mg/m^2^ (3 weeks on, 1 week off) was completed with an upfront 20% dose reduction to ensure tolerance. The CA19-9 remained over >9999 U/mL, and he unfortunately developed biliary and gastric outlet obstruction secondary to the pancreatic primary. He underwent an endoscopic retrograde cholangiopancreatography-guided common bile duct stent insertion for relief of the obstruction. He was also offered a venting G-tube, which he declined in favor of pursuing pharmacologic interventions only. Palliative care was involved after his bowel obstruction, and then intimately through his final months of life, and unfortunately, he passed away 6 weeks after his bowel obstruction with worsening obstructive symptoms and cachexia. All in all, the patient was able to receive four lines of chemotherapy, resulting in a survival of 26 months with minimal toxicity from therapy.

## 4. Discussion

As noted earlier, pancreatic carcinosarcoma is a rare entity that has only been documented in case reports and case series. Previous documentation has used the terms “sarcomatoid carcinoma” and “carcinosarcoma” interchangeably, and thus, clarifying the exact histology discussed in the literature is challenging. Only recently did the 2019 WHO (fifth edition) separate these tumors into distinct variants of undifferentiated ductal adenocarcinoma. Given the rarity of the diagnosis, we conducted a literature search for evidence around sarcomatoid carcinoma, carcinosarcoma, and the broader umbrella classification of undifferentiated carcinoma of the pancreas.

In the latest WHO classification (fifth edition), pancreatic carcinosarcoma is described as a variant of undifferentiated ductal adenocarcinoma, with a characteristic biphasic pattern containing an epithelial component and a distinct sarcomatoid component. By definition, each component should make up approximately 30% of the neoplasm [6,7]. The epithelial component is usually a pancreatic ductal adenocarcinoma (PDAC), ranging from well to poorly differentiated and positive on IHC for epithelial markers (pancytokeratins, CK7, CK19, and BER-EP4). The mesenchymal component may have spindle cells with elongated nuclei, or large, epithelioid cells with prominent nucleoli; rarely, they may show rhabdoid differentiation or include heterologous elements of bone or cartilage, resembling osteosarcoma and chondrosarcoma, respectively. By IHC, the mesenchymal component is negative for cytokeratin and positive for vimentin (Figure 1).

Unlike carcinosarcoma, sarcomatoid undifferentiated carcinomas, another variant of undifferentiated ductal adenocarcinoma, have at least 80% of the neoplasm composed of spindle cells, which co-express epithelial and mesenchymal markers by IHC. These tumors may also include sheets of rhabdoid cells in a myxoid matrix, pleomorphic giant cells, and heterologous elements (bone and cartilage). On IHC, these tumors show a characteristic loss of nuclear expression of INI1 (SMARC1) [8,9].

**Figure 2:**
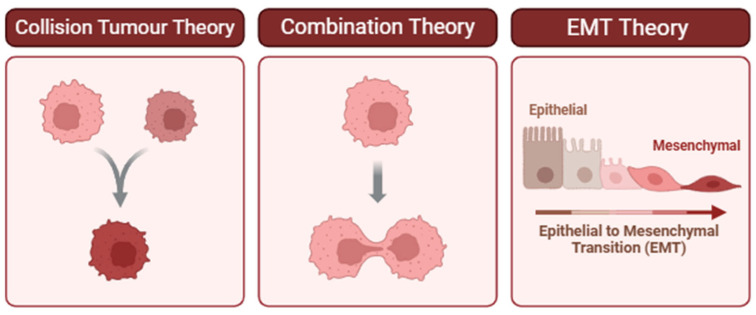
Proposed theories for the pathogenesis of carcinosarcoma. (**Left**) Collision Tumor Theory: Two independent neoplastic clones arise in proximity and ultimately collide, forming a composite tumor mass. This model is supported when distinct histological or molecular profiles are observed in adjacent but genetically unrelated tumor components [11,17]. (**Center**) Combination Theory: A single progenitor cell gives rise to divergent tumor phenotypes through early lineage bifurcation. This theory is supported by shared driver mutations or clonal markers despite differing morphologies [12,13,18]. (**Right**) Epithelial-to-Mesenchymal Transition (EMT) Theory: Epithelial tumor cells undergo EMT, a biological process that enables them to acquire mesenchymal features associated with invasiveness, migration, and therapeutic resistance. Evidence for EMT includes morphological changes and differential expression of markers such as decreased E-cadherin and increased vimentin [19]. Understanding which of these mechanisms predominates in a given tumor can inform diagnostic interpretation and therapeutic decision-making.

Sarcomatoid carcinoma is believed to originate from the epithelial cells of the pancreatic duct, specifically undergoing a process called epithelial–mesenchymal transition (EMT), which transforms the epithelial cells into spindle-shaped cells with mesenchymal characteristics, giving the tumor its sarcomatoid appearance [8,10]. However, the pathogenesis of carcinosarcoma has yet to be conclusively determined, with three main theories as per Figure 2 [6,11,12,13]. The two theories suggesting monoclonal origins—the combination theory and EMT theory—are supported by research showing identical KRAS and TP53 mutations in both carcinosarcoma components [6,14,15,16]. Interestingly, one such study identified co-occurring mutations that are uncommon in PDAC, KRAS Q61H, and TP53 Q100X. The authors therefore posit that specific KRAS or TP53 mutations confer a higher risk of developing carcinosarcomas [14].

As with any rare tumor, definitive pre-operative diagnosis of carcinosarcoma can be challenging, via either imaging or biopsy specimen. It is occasionally possible to distinguish whether a lesion of concern represents a PDAC or one of these rarer histologies based on imaging, such as CT scans. For example, while all three histologies frequently exhibit vascular and perineural invasion, PDAC is less likely to have necrosis, intratumoral calcifications, and hemorrhage [16], and so the visualization of these features on imaging may suggest a non-PDAC tumor. However, it is much more difficult to distinguish between carcinosarcoma and sarcomatoid carcinoma on imaging. Carcinosarcoma may have a more heterogeneous density on CT [16], in keeping with its pathological heterogeneity. Reviews describing sarcomatoid carcinomas showed a higher incidence of solid components and cystic or necrotic components on CT, with well-circumscribed borders, lower propensity for local invasion, and higher propensity for liver and peritoneal dissemination [20,21,22]. However, all differences listed, even those between PDAC and rarer histologies, can be extremely subtle. Biopsy therefore remains significantly more reliable than conventional imaging for distinguishing histology.

On biopsy, heterogenous primaries such as carcinosarcoma may have metastases that are of single-cell origin; a biopsy of one metastasis, even with a large volume sample, may fail to capture the histology of (an)other metastatic site(s). Adequacy and thoroughness of pathological examination can also impact the final diagnosis, as small samples of the minor component can be easily missed. Thus, in situations where the classical histological diagnosis of PDAC does not fit the clinical picture, a biopsy of a different area may be considered. Pathology should be reviewed with pathologists with expertise in gastrointestinal oncology and sarcoma. While circulating tumor DNA (ctDNA) holds promise in the diagnosis of PDAC [23,24], given the rarity of pancreatic carcinosarcoma and the heterogeneity of the tumor, the utility of ctDNA remains unclear.

NGS testing in this case was positive for Tier I/II variants in KRAS (Gly12Val), TP53 (Tyr220Cys), and NF2 (Leu127*). As mentioned above, KRAS mutations and TP53 mutations have previously been identified in both components of carcinosarcoma [6,14,16]. KRAS and TP53 mutations are common in many cancers, including PDAC, where a majority of cases are KRAS-mutated [25]. Sarcomas are common in TP53 mutation carriers, and sarcomas in non-germline mutation carriers often have TP53 alterations [26,27]. NF2 alterations are characteristic of schwannomas and meningiomas and appear to occur much more rarely in other cancers [28]. One recently published report on carcinosarcoma of the uterus identified an NF2 mutation alongside an ATM mutation; further information on the prevalence of NF2 in carcinosarcoma is needed [29]. Sending carcinosarcoma samples for NGS panel testing is recommended and may provide critical information for future study.

In this case, CA19-9 was tracked throughout the treatment course. CA19-9 has long been validated as a useful biomarker for diagnosis and response monitoring of PDAC, given its sensitivity of 80–95% and specificity of 80–90% [30,31,32]. However, it is not secreted by all PDAC. CA19-9 is also useful as a prognostic marker, as a high pre-operative level suggests a worse surgical outcome [30,33,34,35,36]. However, in carcinosarcoma, CA19-9 levels have not been found to consistently correlate with disease burden [6,16,37,38]. Our patient’s CA19-9 levels were both concordant with the disease burden and useful in tracking disease response, a unique feature of this case. This may suggest a more predominant component of adenocarcinoma or perhaps support the single-cell origin theory in this patient’s carcinosarcoma. It is worth assessing in each case of carcinosarcoma whether CA19-9 correlates with response.

For staging of metastatic pancreatic malignancies, dedicated CT or MRI pancreas scans with CT chest, abdomen, and pelvis scans remain the standard radiographic tools [34]. Positron emission tomography (PET) scans are not a routine part of pancreatic cancer management [39], as local imaging findings overlap with those of autoimmune and chronic pancreatitis, and it has not been proven superior to CT scans in the identification of distant metastases [40,41]. Interestingly, a 2020 single-center retrospective review of four patients who underwent PET scan for pancreatic sarcomatoid carcinoma demonstrated an average SUVmax of 16 and 22 in the early and delayed phase, respectively, which is much higher than the historical average of 3-9 for PDAC [42,43]. The utility of PET scan in the diagnosis and staging of carcinosarcoma remains unclear and should be considered on a case-by-case basis. The role of fibroblast activation protein PET (FAP-PET) in pancreatic carcinosarcoma is an emerging area of interest, given the tumor’s aggressive nature and the known limitations of conventional imaging modalities [44].

A major challenge in systemically treating carcinosarcoma is selecting a regimen that can effectively control both histologies, as neither carries a good prognosis if left untreated. The two currently accepted first-line treatments for advanced PDAC are gemcitabine and nab-paclitaxel, with a median overall survival (mOS) of 8.5 months [45], and FOLFIRINOX, with a mOS of 11.1 months at the cost of greater toxicity [46]. Currently, there is no standard first-line treatment for primary soft-tissue sarcoma (STS) of the pancreas. Anthracyclines are considered one of the most effective agents in STS generally, with an objective response rate of 11–25% [47,48]. Other effective chemotherapy agents include single-agent gemcitabine or a gemcitabine doublet with docetaxel, vinorelbine, or dacarbazine [49]. A recent multi-center retrospective review of 50 patients with undifferentiated carcinoma of the pancreas, a broader WHO classification which includes carcinosarcoma and sarcomatoid carcinoma, demonstrated a 33% response rate with gemcitabine and nab-paclitaxel and a 4.6-month progression-free survival. In this study, only four patients (8%) had confirmed sarcomatoid carcinoma, while 32% had anaplastic histology and 38% had undifferentiated carcinoma, not otherwise specified [50]. Based on past demonstrated efficacy across both histologies, gemcitabine and nab-paclitaxel were felt to be the most appropriate choice of first-line treatment.

The prevalence of high microsatellite instability (MSI-H) in pancreatic sarcomatoid carcinoma is not well-documented, likely due to the rarity of the disease. Genomic profiling of undifferentiated pancreatic sarcomatoid carcinoma suggests higher PD-L1 positivity on IHC in comparison to non-sarcomatoid carcinoma, with a small proportion (5%) being MSI-H/high tumor mutational burden. One case report of MSI-high metastatic pancreatic sarcomatoid carcinoma demonstrated prolonged survival with later-line pembrolizumab [51]. Unfortunately, our patient had MMR-proficient disease with no targetable mutation on next-generation sequencing and could not utilize these specialized options.

To our knowledge, this is the first case report to document the long-term chemotherapeutic management of de novo metastatic pancreatic carcinosarcoma using multiple lines of therapy. Past case reports have either described early/localized disease, which allowed for definitive surgery and potentially neoadjuvant or adjuvant chemotherapy, or those with symptomatic advanced disease who were too frail to receive or declined treatment. A summary of pancreatic carcinosarcoma cases where chemotherapy was utilized can be found in Table 1. There are two reports documenting the potential benefit of chemotherapy in metastatic pancreatic carcinosarcoma. One discovered metastatic disease during adjuvant gemcitabine and nab-paclitaxel and switched to second-line FOLFOX (leucovorin, fluorouracil, oxaliplatin) when CA19-9 remained persistently elevated despite no imaging progression [52]. After 9 cycles of first-line and 11 cycles of second-line therapy, the patient passed away from disease progression 16 months post-operatively. The second describes a patient with a *BRCA1* germline mutation who showed sustained complete response after 12 cycles of FOLFIRINOX, initially given adjuvantly and continued when liver metastases were diagnosed within one cycle of chemotherapy initiation [53]. The patient’s *BRCA1*-mutated status, which can indicate improved survival with platinum-containing chemotherapy, may impact generalizability but does support the use of FOLFIRINOX in this setting. Our patient was remarkable in his fitness and lack of comorbidities at his age, which enabled him to withstand multiple lines of systemic chemotherapy. Unfortunately, due to the rarity of this cancer, he was not qualified for any clinical trials. Despite the lack of trial data and his fitness, it was still exceptional to achieve nearly two years of survival with this disease diagnosed at such an advanced stage.

## 5. Conclusions

Pancreatic carcinosarcoma is a rare and aggressive cancer with a poor prognosis regardless of stage. While there is no standard of care treatment due to the rarity of the disease, a multi-disciplinary approach is critical in the management of these cases, and even in the advanced setting, conventional chemotherapy for pancreatic adenocarcinoma may help improve survival in select cases. This patient presented here achieved an unprecedented overall survival of 26 months with good quality of life for the majority of this time via four lines of chemotherapy: first-line gemcitabine and nab-paclitaxel, second-line modified FOLFIRINOX, third-line GTX, and fourth-line doxorubicin. This regimen and sequencing may be utilized in fit patients with similar diagnoses. Finally, a consideration of comprehensive NGS testing and clinical trial enrollment should be considered where applicable.

## Figures and Tables

**Table 1:** Case reports of pancreatic carcinosarcoma treated with chemotherapy. Summaries of relevant literature cases with citations are included above that note all cases where patients were treated with chemotherapy in any setting, namely adjuvant, neoadjuvant, and metastatic. Column 2 contains a summary of the therapeutic actions taken and any unique features. Column 3 contains the reported pathologic and IHC information. Column 4 summarizes case outcomes.

Report	Relevant Case Details	Relevant Pathology Findings	Outcome
Gelos et al., 2008 [54]	Resected. Adjuvant gemcitabine, 6 cycles.	Moderately differentiated adenocarcinoma and poorly differentiated solid tissue. IHC epithelial: strongly positive CK7 and pan-CK, moderate positive CK20. IHC sarcomatoid: strongly positive vimentin; negative for all CK antibodies.	Recurrence 11 months post-operatively, relaparotomy, death 2 days later.
Kim et al., 2011 [15]	Resected. Adjuvant gemcitabine, progressed after 3 cycles.	Anaplastic carcinoma and sarcomatoid component in solid lesion. Additional cystic lesion. IHC anaplastic: strongly positive EMA, pan-CK, CK7, CK8/18, and monoclonal/polyclonal CEA; 90% p53 positive. IHC sarcomatoid: strong positive vimentin; negative pan-CK, CK7, CK8/18, alpha-1-antitrypsin, alpha-1-antichymotrypsin, CD34, CD56, CD68, CD117, desmin, estrogen receptor, progesterone receptor, human melanoma black 45 (HMB45), lysozyme, myogenin, S100 and SMA; 80% p53 positive.	Death 4 months post-resection.
Zhu et al., 2011 [55]	Resected. Adjuvant gemcitabine–doxorubicin–cisplatin, 5 cycles.	Ductal carcinoma and sarcoma. IHC carcinoma: strongly positive CK18, EMA. IHC sarcomatous: strongly positive SMA; negative CK18, EMA, S-100.	No recurrence 20 months post-op.
Jia et al., 2017 [56]	Resected. Adjuvant gemcitabine–raltitrexed, 8 cycles.	Moderately differentiated adenocarcinoma and heterologous mesenchymal osteosarcoma. IHC positive CK7, vimentin.	No recurrence 31 months post-op.
Salibay et al., 2017 [57]	Surgical biopsy, no resection. First-line gemcitabine–docetaxel, no response. Second-line doxorubicin–ifosfamide, progressive disease. Plan for palliative RT.	High-grade spindle cell sarcoma and moderately differentiated adenocarcinoma. IHC adenocarcinoma: positive CKAE1/3, CK7, villin, focal CDX2; negative CD10, CK20, desmin, ER, myogenin, TTF1; Ki67 50%. IHC sarcoma: strongly positive CD10; focally positive desmin, SMA; negative CKAE1/3, ER, myogenin, villin, DOG1, CD117; Ki67 90%.	Death 10 months post- diagnosis.
Still et al., 2018 [58]	Initial biopsy showed adenocarcinoma. Neoadjuvant FOLFIRINOX, 3 months. Resected. Adjuvant gemcitabine–paclitaxel, metastatic disease identified 1 month post-op.	60% sarcoma with focal chondrosarcoma and myogenic differentiation, 40% moderately differentiated adenocarcinoma. IHC: patchy positive CK, desmin; nonspecific staining myoglobin; negative myogenin, myo-D1.	Death 13 months post-diagnosis.
Li et al., 2020 [14]	Resected. Adjuvant gemcitabine–paclitaxel.	Disparate carcinomatous and sarcomatous components. IHC carcinoma: positive EMA and CK7/8; negative vimentin IHC sarcoma: positive vimentin; negative EMA and CK7/8. Sarcoma components had higher Ki67 than carcinoma components.	Recurred 3 months post-op. Death 11 months post-op.
	Resected. Adjuvant mFOLFIRINOX.		Recurred 13 months post-op. Death 19 months post-op.
	Resected. Adjuvant gemcitabine.		Recurred 10 months post-op. Death 17 months post-op.
Quinn et al., 2020 [52]	Initial biopsy, inconclusive. Resected. Adjuvant gemcitabine–paclitaxel, 9 cycles total, liver metastases discovered 2 months post-op. Second-line FOLFOX, completed 11 cycles.	Glandular areas and stroma containing atypical spindle cells; labeled mixed mucinous adenocarcinoma and heterologous anaplastic sarcomatous components. IHC epithelial: positive SMA. IHC sarcomatous: positive CD31.	Discontinuation of therapy 16 months post-op and transition to comfort care.
Lalonde et al., 2022 [59]	Identified as borderline resectable. Neoadjuvant FOLFIRINOX, 4 months. 2 months concurrent chemoradiotherapy with capecitabine. Resection.	Invasive poorly differentiated carcinosarcoma of the pancreas with both glandular and sarcomatoid components	No recurrence 15 months post-op.
Lee et al., 2023 [38]	Initial biopsy. 11 cycles neoadjuvant FOLFIRINOX-pembrolizumab (trial); SBRT 5 fractions; robotic resection. Adjuvant FOLFIRINOX.	Biopsy: adenocarcinoma with irregular glands, poorly differentiated sarcoma. IHC epithelial: strongly positive CK. IHC sarcoma: strongly positive vimentin.	No recurrence 7 months post-op.
Gilani et al., 2025 [53]	BRCA1 mutation. Resected. Planned adjuvant FOLFIRINOX. Liver metastases found on imaging 1 month into therapy.	Poorly differentiated carcinosarcoma, grade 2. Specific pathological details not provided.	Long-term complete response in liver with 12 cycles FOLFIRINOX.

## Data Availability

The original contributions presented in this study are included in the article. Further inquiries can be directed to the corresponding author.

## Abbreviations

The following abbreviations are used in this manuscript:

CT	computed tomography
ctDNA	circulating tumor DNA
EMT	epithelial–mesenchymal transition
EUS	endoscopic ultrasound
FOLFIRINOX	leucovorin, 5-fluorouracil, irinotecan, oxaliplatin
FOLFOX	leucovorin, 5-fluorouracil, oxaliplatin
GTX	gemcitabine, docetaxel, capecitabine
IHC	immunohistochemistry
MMR	mismatch repair
mOS	median overall survival
MSI-H	high microsatellite instability
NGS	next-generation sequencing
PDAC	pancreatic ductal adenocarcinoma
PET	positron emission tomography
SABR	stereotactic ablative radiotherapy
SMA	superior mesenteric artery
RT	radiation
WHO	World Health Organization

## References

[1] Alhatem A., Quinn P.L., Xia W., Chokshi R.J. (2021). Pancreatic Carcinosarcoma Clinical Outcome Analysis of the National Cancer Institute Database. J. Surg. Res..

[2] Nagtegaal I.D., Odze R.D., Klimstra D., Paradis V., Rugge M., Schirmacher P., Washington K.M., Carneiro F., Cree I.A. (2020). The 2019 WHO classification of tumours of the digestive system. Histopathology.

[3] Masuda T., Dann A.M., Elliott I.A., Baba H., Kim S., Sedarat A., Muthusamy V.R., Girgis M.D., Hines O.J., Reber H.A. (2018). A Comprehensive Assessment of Accurate Lymph Node Staging and Preoperative Detection in Resected Pancreatic Cancer. J. Gastrointest. Surg..

[4] Kolbeinsson H., Hoppe A., Bayat A., Kogelschatz B., Mbanugo C., Chung M., Wolf A., Assifi M.M., Wright G.P. (2021). Recurrence patterns and postrecurrence survival after curative intent resection for pancreatic ductal adenocarcinoma. Surgery.

[5] Li Q., Feng Z., Miao R., Liu X., Liu C., Liu Z. (2022). Prognosis and survival analysis of patients with pancreatic cancer: Retrospective experience of a single institution. World J. Surg. Oncol..

[6] Ruess D.A., Kayser C., Neubauer J., Fichtner-Feigl S., Hopt U.T., Wittel U.A. (2017). Carcinosarcoma of the Pancreas: Case Report With Comprehensive Literature Review. Pancreas.

[7] Khan J., Cheng L., House M.G., Guo S. (2021). Carcinosarcoma, a Rare Malignant Neoplasm of the Pancreas. Curr. Oncol..

[8] Gkountakos A., Simbolo M., Bariani E., Scarpa A., Luchini C. (2022). Undifferentiated Sarcomatoid Carcinoma of the Pancreas: From Histology and Molecular Pathology to Precision Oncology. Int. J. Mol. Sci..

[9] Ma Y., Yang Y., Zhang H., Mugaanyi J., Hu Y., Wu S., Lu C., Mao S., Wang K. (2024). Sarcomatoid carcinoma of the pancreas (Review). Oncol. Lett..

[10] Zhou P., Li B., Liu F., Zhang M., Wang Q., Liu Y., Yao Y., Li D. (2017). The epithelial to mesenchymal transition (EMT) and cancer stem cells: Implication for treatment resistance in pancreatic cancer. Mol. Cancer.

[11] Miyauchi J., Ogura M., Sato M., Matsui J. (2018). Esophageal carcinosarcoma comprised of minimally invasive squamous cell carcinoma and undifferentiated pleomorphic sarcoma: A collision cancer?. Pathol. Int..

[12] Matsumoto T., Fujii H., Arakawa A., Yamasaki S., Sonoue H., Hattori K., Kajiyama Y., Hirose S., Tsurumaru M. (2004). Loss of heterozygosity analysis shows monoclonal evolution with frequent genetic progression and divergence in esophageal carcinosarcoma. Hum. Pathol..

[13] Gotoh O., Sugiyama Y., Takazawa Y., Kato K., Tanaka N., Omatsu K., Takeshima N., Nomura H., Hasegawa K., Fujiwara K. (2019). Clinically relevant molecular subtypes and genomic alteration-independent differentiation in gynecologic carcinosarcoma. Nat. Commun..

[14] Li J., Wei T., Zhang J., Wei S., Chen Q., Chen B.W., Zhou Y., Wen L., Qin H., Bai X. (2020). Carcinosarcoma of the pancreas: Comprehensive clinicopathological and molecular characterization. HPB.

[15] Kim H.-S., Joo S.H., Yang D.M., Lee S.H., Choi S.H., Lim S.J. (2011). Carcinosarcoma of the Pancreas: A Unique Case with Emphasis on Metaplastic Transformation and the Presence of Undifferentiated Pleomorphic High-Grade Sarcoma. J. Gastrointestin. Liver Dis..

[16] Bai Q., Zhang X., Zhu X., Wang L., Huang D., Cai X., Zhou X., Wang J., Sheng W. (2016). Pancreatic carcinosarcoma with the same KRAS gene mutation in both carcinomatous and sarcomatous components: Molecular evidence for monoclonal origin of the tumour. Histopathology.

[17] Zalewski A., Chlebicka I., Szepietowski J.C. (2024). Collision tumours: Our recent experience. Postępy Dermatol. Alergol..

[18] Petersson F. (2015). Mixed tumors and collision tumors: A unifying concept with relevance to diagnosis and classification. Path. Int..

[19] Nieto M.A., Huang R.Y., Jackson R.A., Thiery J.P. (2016). EMT: 2016. Cell.

[20] Shi H.-Y., Xie J., Miao F. (2015). Pancreatic carcinosarcoma: First literature report on computed tomography imaging. World J. Gastroenterol..

[21] Lim H.J., Kang H.S., Lee J.E., Min J.H., Shin K.S., You S.K., Kim K.H. (2021). Sarcomatoid carcinoma of the pancreas—Multimodality imaging findings with serial imaging follow-up: A case report and review of literature. World J. Clin. Cases.

[22] Zhao S., Su W., Deng L., Chen Y., Zuo C., Shao C., Ren F. (2020). Pancreatic sarcomatoid carcinoma: CT, MRI, and 18F-FDG PET/CT features. Clin. Radiol..

[23] Mencel J., Feber A., Begum R., Carter P., Smalley M., Bourmpaki E., Shur J., Zar S., Kohoutova D., Popat S. (2022). Liquid biopsy for diagnosis in patients with suspected pancreatic and biliary tract cancers: PREVAIL ctDNA pilot trial. J. Clin. Oncol..

[24] Grunvald M.W., Jacobson R.A., Kuzel T.M., Pappas S.G., Masood A. (2020). Current Status of Circulating Tumor DNA Liquid Biopsy in Pancreatic Cancer. Int. J. Mol. Sci..

[25] Jones S., Zhang X., Parsons D.W., Lin J.C., Leary R.J., Angenendt P., Mankoo P., Carter H., Kamiyama H., Jimeno A. (2008). Core signaling pathways in human pancreatic cancers revealed by global genomic analyses. Science.

[26] Wang Y., Min L., Zhou Y., Tang F., Luo Y., Zhang W., Duan H., Tu C. (2019). The efficacy and safety of apatinib in metastatic alveolar soft part sarcoma: A case series of six patients in one institution. Cancer Manag. Res..

[27] Ognjanovic S., Olivier M., Bergemann T.L., Hainaut P. (2012). Sarcomas in TP53 germline mutation carriers: A review of the IARC TP53 database. Cancer.

[28] Xu D., Yin S., Shu Y. (2024). NF2: An underestimated player in cancer metabolic reprogramming and tumor immunity. NPJ Precis. Oncol..

[29] Irie T., Iida Y., Hamada Y., Matsushima J., Iizuka M., Takakura S. (2025). Primary carcinosarcoma of the uterine cervix with somatic mutations of the *ATM* and *NF2* genes: A case report. Int. Cancer Conf. J..

[30] Azizian A., Rühlmann F., Krause T., Bernhardt M., Jo P., König A., Kleiß M., Leha A., Ghadimi M., Gaedcke J. (2020). CA19-9 for detecting recurrence of pancreatic cancer. Sci. Rep..

[31] Wlodarczyk B., Durko L., Wlodarczyk P., Talar-Wojnarowska R., Malecka-Wojciesko E. (2023). CA 19-9 but Not IGF-1/IGFBP-2 Is a Useful Biomarker for Pancreatic Ductal Adenocarcinoma (PDAC) and Chronic Pancreatitis (CP) Differentiation. J. Clin. Med..

[32] Zhao B., Zhao B., Chen F. (2022). Diagnostic value of serum carbohydrate antigen 19-9 in pancreatic cancer: A systematic review and meta-analysis. Eur. J. Gastroenterol. Hepatol..

[33] Asaoka T., Miyamoto A., Maeda S., Tsujie M., Hama N., Yamamoto K., Miyake M., Haraguchi N., Nishikawa K., Hirao M. (2016). Prognostic impact of preoperative NLR and CA19-9 in pancreatic cancer. Pancreatology.

[34] Takagi C., Kikuchi Y., Shirakawa H., Hoshimoto S., Tomikawa M., Ozawa I., Hishinuma S., Ogata Y. (2019). Predictive Factors for Elevated Postoperative Carbohydrate Antigen 19-9 Levels in Patients With Resected Pancreatic Cancer. Anticancer Res..

[35] Dong Q., Yang X.H., Zhang Y., Jing W., Zheng L.Q., Liu Y.P., Qu X.J. (2014). Elevated serum CA19-9 level is a promising predictor for poor prognosis in patients with resectable pancreatic ductal adenocarcinoma: A pilot study. World J. Surg. Oncol..

[36] Bergquist J.R., Puig C.A., Shubert C.R., Groeschl R.T., Habermann E.B., Kendrick M.L., Nagorney D.M., Smoot R.L., Farnell M.B., Truty M.J. (2016). Carbohydrate Antigen 19-9 Elevation in Anatomically Resectable, Early Stage Pancreatic Cancer Is Independently Associated with Decreased Overall Survival and an Indication for Neoadjuvant Therapy: A National Cancer Database Study. J. Am. Coll. Surg..

[37] Kim H.S., Kim J.I., Jeong M., Seo J.H., Kim I.K., Cheung D.Y., Kim T.J., Kang C.S. (2014). Pancreatic adenocarcinosarcoma of monoclonal origin: A case report. World J. Gastroenterol..

[38] Lee M., Cho Y.J., Jung H.S., Yun W.G., Han Y., Kwon W., Jang J.Y. (2023). Collective review of pancreatic carcinosarcoma, a very rare pancreatic malignancy. Ann. Hepatobiliary Pancreat. Surg..

[39] Conroy T., Pfeiffer P., Vilgrain V., Lamarca A., Seufferlein T., O’Reilly E.M., Hackert T., Golan T., Prager G., Haustermans K. (2023). Pancreatic cancer: ESMO Clinical Practice Guideline for diagnosis, treatment and follow-up. Ann. Oncol..

[40] Wu L.M., Hu J.N., Hua J., Liu M.J., Chen J., Xu J.R. (2012). Diagnostic value of diffusion-weighted magnetic resonance imaging compared with fluorodeoxyglucose positron emission tomography/computed tomography for pancreatic malignancy: A meta-analysis using a hierarchical regression model. J. Gastroenterol. Hepatol..

[41] Wang L., Dong P., Wang W.G., Tian B.L. (2017). Positron emission tomography modalities prevent futile radical resection of pancreatic cancer: A meta-analysis. Int. J. Surg..

[42] Liu X., Ren Y., Wang J., Yang X., Lu L. (2022). The Clinical Diagnostic Value of F-FDG PET/CT Combined with MRI in Pancreatic Cancer. Contrast Media Mol. Imaging.

[43] Zhang J., Jia G., Zuo C., Jia N., Wang H. (2017). 18F- FDG PET/CT helps differentiate autoimmune pancreatitis from pancreatic cancer. BMC Cancer.

[44] Dendl K., Koerber S.A., Kratochwil C., Cardinale J., Finck R., Dabir M., Novruzov E., Watabe T., Kramer V., Choyke P.L. (2021). FAP and FAPI-PET/CT in Malignant and Non-Malignant Diseases: A Perfect Symbiosis?. Cancers.

[45] Von Hoff D.D., Ervin T., Arena F.P., Chiorean E.G., Infante J., Moore M., Seay T., Tjulandin S.A., Ma W.W., Saleh M.N. (2013). Increased Survival in Pancreatic Cancer with nab-Paclitaxel plus Gemcitabine. N. Engl. J. Med..

[46] Orlandi E., Citterio C., Anselmi E., Cavanna L., Vecchia S. (2024). FOLFIRINOX or Gemcitabine Plus Nab-paclitaxel as First Line Treatment in Pancreatic Cancer: A Real-World Comparison. Cancer Diagn. Progn..

[47] Kciuk M., Gielecińska A., Mujwar S., Kołat D., Kałuzińska-Kołat Ż., Celik I., Kontek R. (2023). Doxorubicin-An Agent with Multiple Mechanisms of Anticancer Activity. Cells.

[48] Sritharan S., Sivalingam N. (2021). A comprehensive review on time-tested anticancer drug doxorubicin. Life Sci..

[49] Gurruchaga S.I., Gómez-Mateo M.C., Ortega Izquierdo M.E., Martínez-Trufero J. (2024). Beneficial Use of the Combination of Gemcitabine and Dacarbazine in Advanced Soft Tissue Sarcomas: Real-World Data. Cancers.

[50] Imaoka H., Ikeda M., Maehara K., Umemoto K., Ozaka M., Kobayashi S., Terashima T., Inoue H., Sakaguchi C., Tsuji K. (2020). Clinical outcomes of chemotherapy in patients with undifferentiated carcinoma of the pancreas: A retrospective multicenter cohort study. BMC Cancer.

[51] Mayrhofer K. (2021). Pembrolizumab in MSI-high Pancreatic Sarcomatoid Carcinoma. Ann. Hematol. Oncol..

[52] Quinn P.L., Ohioma D., Jones A.M.K., Ahlawat S.K., Chokshi R.J. (2020). Treatment of Rare and Aggressive Pancreatic Carcinosarcoma. ACG Case Rep..

[53] Gilani S., Ibrahim M.A., Mujeeb Q., Khir I. (2025). Pancreatic carcinosarcoma: A rare type of pancreatic neoplasia with long-term survival. BMJ Case Rep..

[54] Gelos M., Behringer D., Philippou S., Mann B. (2008). Pancreatic carcinosarcoma. Case report of multimodal therapy and review of the literature. J. Pancreas.

[55] Zhu W.Y., Liu T.G., Zhu H. (2012). Long-term recurrence-free survival in a patient with pancreatic carcinosarcoma: A case report with a literature review. Med. Oncol..

[56] Jia Z., Zhang K., Huang R., Zhou X., Jiang L. (2017). Pancreatic carcinosarcoma with rare long-term survival: Case report and review of the literature. Medicine.

[57] Salibay C.J., Rewerska J., Gupta S., Ree N. (2017). Primary Carcinosarcoma of the Pancreas With CD10-Positive Sarcoma Component. J. Investig. Med. High Impact Case Rep..

[58] Still S.A., Becerra C.R., Clement-Kruzel S.E., Cavaness K.M. (2018). Locally advanced carcinosarcoma of the pancreas. Bayl. Univ. Med. Cent. Proc..

[59] Lalonde C.S., Wang L., Quigley B., Patel P., Maithel S.K., El-Rayes B.F., Akce M. (2022). Neoadjuvant treatment of pancreatic carcinosarcoma: A case report and review of literature. Chin. Clin. Oncol..

